# Associations Between Subjective Tinnitus and Cognitive Performance: Systematic Review and Meta-Analyses

**DOI:** 10.1177/2331216520918416

**Published:** 2020-05-21

**Authors:** Nathan A. Clarke, Helen Henshaw, Michael A. Akeroyd, Bethany Adams, Derek J. Hoare

**Affiliations:** 1 NIHR Nottingham Biomedical Research Centre; 2 Division of Clinical Neuroscience, School of Medicine, University of Nottingham

**Keywords:** tinnitus, cognition, cognitive performance, attention, memory, executive function

## Abstract

Tinnitus is the perception of sound in the absence of a corresponding external sound source, and bothersome tinnitus has been linked to poorer cognitive performance. This review comprehensively quantifies the association between tinnitus and different domains of cognitive performance. The review protocol was preregistered and published in a peer-reviewed journal. The review and analyses were reported according to Preferred Reporting Items for Systematic Review and Meta-analysis guidelines. Peer-reviewed literature was searched using electronic databases to find studies featuring participants with tinnitus who had undertaken measures of cognitive performance. Studies were assessed for quality and categorized according to an established cognitive framework. Random-effects meta-analyses were performed on various cognitive domains with potential moderator variables assessed where possible. Thirty-eight records were included in the analysis from a total of 1,863 participants. Analyses showed that tinnitus is associated with poorer executive function, processing speed, general short-term memory, and general learning and retrieval. Narrow cognitive domains of Inhibition and Shifting (within executive function) and learning and retrieval (within general learning and retrieval) were also associated with tinnitus.

Tinnitus is the perception of sound in the absence of a corresponding external sound source. Objective tinnitus refers to perceived sound that is caused by an identified internal mechanism, such as turbulent blood flow. However, most cases of tinnitus have no identifiable causes and so are described as subjective. Approximately 10% to 15% of the population are thought to experience tinnitus, with 5% of these being described as having clinically significant or bothersome tinnitus. Bothersome tinnitus is associated with depression and anxiety, and symptoms include sleep disruption and concentration difficulties (Hall et al., 2018; Watts et al., 2018).

It is essential to understand concentration difficulties related to tinnitus because it is a very common complaint and significantly contributes to the construct of self-reported tinnitus severity (Fackrell et al., 2016). Mohamad et al. (2016) note that while the term concentration is typically a layperson’s label for their personal experience, cognition can be studied scientifically through task performance across different domains, such as attention, memory, and executive functioning. Cognitive constructs are typically measured through task performance assessing response times and accuracy rates. Cognitive performance can also be self-reported through questionnaires. Although it has been suggested that tinnitus impacts cognitive performance through disrupting executive attention (Heeren et al., 2014; Tegg-Quinn et al., 2016), an improved understanding of cognitive performance in participants with tinnitus has the potential to provide corroborating functional evidence for observed brain structures implicated in tinnitus generation and maintenance. This is particularly important given the role that attention plays in many prominent theories of tinnitus generation and its subsequent maintenance (Roberts et al., 2013; Sedley et al., 2016). Furthermore, there is considerable research interest in training cognitive abilities (Diamond & Ling, 2016), with training of associated cognitive domains representing a promising yet unexplored means of therapeutic management, both for amelioration of concentration difficulties and for the impact of such training on the tinnitus percept. It is important to understand which cognitive domains are associated with tinnitus before an effective investigation of potential therapies can begin.

Many reviews have discussed the importance of cognition in tinnitus (Andersson & McKenna, 2006; Mohamad et al., 2016; Schultz et al., 2018; Tavanai & Mohammadkhani, 2018; Tegg-Quinn et al., 2016; Trevis et al., 2018). Two previous reviews have concluded that tinnitus causes poorer cognitive performance through disrupting “executive attention” (Mohamad et al., 2016; Tegg-Quinn et al., 2016), which is described by Mohamad et al. (2016, pp. 3–4) as “resolving input by engaging, disengaging, and switching attention.” Previous reviews have been primarily narrative and typically reported difficulties synthesizing results due to seemingly equivocal evidence; they have also featured different subsets of previous studies, essentially reviewing differing sets of evidence. Furthermore, with the exception of executive attention, previous reviews have summarized the relationship between tinnitus and cognitive performance in various domains, such as working memory, as having mixed results (i.e., statistically significant or not), potentially leaving researchers and clinicians uncertain as to whether tinnitus is associated with poorer cognitive performance. The findings of previous reviews therefore highlight the difficulties in drawing firm conclusions about the current evidence solely through narrative synthesis, which has noted shortcomings such as potentially facilitating author bias, opacity of methods and quality assessment, and questionable comprehensiveness (Ellis, 2010; Quintana, 2015).

Here, we present the results of a preregistered systematic review and meta-analyses using bias-limiting methods to reduce the influence of “researcher degrees of freedom,” where arbitrary substantive or methodological decisions compound to achieve a final “significant” result (Wicherts et al., 2016). This is the first review to apply a cognitive taxonomy to connect theorizing regarding tinnitus and cognitive performance to current theories within cognitive psychology. The aim was to undertake a comprehensive systematic review and meta-analyses, to answer the question: “Is subjective tinnitus associated with poorer cognitive performance?” In answering this question, we use an established cognitive framework (Webb et al., 2018) to analyze:

- Which broad domains of cognition are associated with tinnitus
- Which narrow domains of cognition are associated with tinnitus
- How strong the associations are between tinnitus and performance in various cognitive domains
- What the quality of the available evidence is
- Which moderating variables confound any identified associations between cognitive performance and tinnitus

## Methods

The review is reported according to Preferred Reporting Items for Systematic Review and Meta-Analysis (Moher et al., 2009). The review protocol was preregistered on the PROSPERO database (CRD42018085528) and published in a peer-reviewed journal (Clarke et al., 2018).

### Databases and Search Strategy

Electronic searches for peer-reviewed journal articles were performed in MEDLINE (via Ovid SP), EMBASE (via Ovid SP), PsycINFO (via Ovid SP), ASSIA (via ProQuest), Cumulative Index to Nursing and Allied Health Literature or CINAHL (via EBSCO Host), Scopus, PubMed, and Web of Science (Science and Social Science Citation Index). Gray literature including PhD theses and conference proceedings were eligible for inclusion. However, all studies identified in gray literature were subsequently matched to peer-reviewed publication. We therefore reported the peer-reviewed version of the study record only.

Search terms entered into the databases were identified using free text, controlled vocabularies (e.g., Medical subject headings and CINAHL Headings), literature review, opinion of authors, and scrutiny of pilot search results. A record of search activity and the relevant search strings for each database is provided as Supplementary Materials. Hand searches of key journals (identified via electronic database searches and author discretion) were also performed. Searches were conducted in February 2018 and updated in January 2019.

### Inclusion Criteria

Published or in-press studies, written in English or with an available English translation, were eligible for inclusion. No date restrictions were applied to searches. Review inclusion criteria were specified according to Participant, Intervention/Interest, Comparator, Outcome, and Setting:

#### Participants

Studies including adults (≥18 years) with tinnitus. Studies that included both children (<18 years) and adults were excluded, unless the adult data were reported separately.

#### Intervention/Interest

Studies including participants with self-reported tinnitus.

#### Comparator

Studies reporting at least one established measure of cognitive performance (behavioral or self-report).

#### Outcome Measures

Studies reporting a measure of association between tinnitus and cognitive performance or studies containing potential requisite descriptive data to calculate an association. Where available, data for associations between tinnitus and additional potential moderator variables, such as measures of anxiety or depression, were also extracted.

#### Study Design

Cross-sectional, longitudinal, experimental, quasi-experimental, and observational studies were included. For studies where multiple time-point measurements were made, baseline data were extracted.

### Article Screening and Selection

Article screening and record management were performed using Covidence systematic review software (https://www.covidence.org). Titles and abstracts of records were independently screened by at least two members of the review team and discrepancies discussed. Records required consensus by both reviewers to be taken forward for full-text review. A third member of the review team adjudicated in instances where consensus was not obtained between initial reviewers. Articles were then taken forward to full-text screening, including articles where there was too little information to make an initial decision via title and abstract screening. Included records were those that met the inclusion criteria following full-text review.

### Data Extraction and Data Items

Included records were subjected to data extraction using a data extraction form developed by the review team. Each record was subject to data extraction independently by two members of the review team. Extracted data items were then compared across reviewers for accuracy and consistency. In instances where extracted data items differed for a record, discussion and comparison to clarify extracted data were undertaken between the reviewers.

### Weight of Evidence—Record Bias and Quality

Records were rated according to their relevant risk of bias and study quality using a weight of evidence (WoE) framework (Gough, 2007; Weed, 2005). A WoE tool was devised by the review team (Appendix), and each record was rated independently by two reviewers. In instances where WoE ratings between reviewers did not agree, discussion was undertaken to clarify rating rationale until consensus was achieved. WoE ratings between members of the review team displayed excellent agreement with two thirds of ratings achieving complete agreement on initial comparison. Complete agreement for all quality ratings was attained following initial clarification and discussion of ratings, with no need for third author adjudication.

### Calculation of Effect Sizes

The effect size of interest was the product-moment correlation (*r*). For records where measures of association between cognitive tasks and tinnitus severity were not reported, an estimate of the effect size was calculated from available descriptive statistics. The variety of study designs included in the review meant that available data necessary to calculate estimates of effect sizes differed across records. Estimates of the correlation coefficient between tinnitus and cognitive performance were derived from different descriptive statistics, dependent on what data were available. The correlation coefficient was chosen for its noted flexibility as an effect size metric (McGrath & Meyer, 2006), allowing for a less selective data synthesis strategy.

- If a record reported a single Pearson correlation per measure of cognitive performance the correlation was used, and the data item was coded as reported.
- If a record reported multiple tinnitus measurements and multiple Pearson correlations per measure of cognitive performance, then the correlation between the primary tinnitus questionnaire and measure of cognitive performance was used, and the data item was coded as reported.
- If the record did not report a Pearson correlation between tinnitus questionnaires and cognitive performance, then the data item was coded as not reported.

A Pearson correlation was calculated from available descriptive and test statistics using an effect-size calculator (http://www.campbellcollaboration.org/escalc/html/EffectSizeCalculator-Home.php). If a record did not report sufficient descriptive statistics to derive a Pearson correlation, then a standardized mean difference (SDM) was calculated (Lipsey & Wilson, 2000, p. 198). SDMs were converted to point-biserial correlations (pbr) using the formula (Polanin & Snilstveit, 2016):

$$r=\frac{d}{\sqrt{d^{2}+a}},$$

where *r* is the correlation coefficient, *d* is the SDM, and *a* is given by

$$a=\frac{{\left(n_{1}+n_{2}\right)}^{2}}{n_{1}\times n_{2}},$$

where *n* is the size of each group.

For some records, relevant statistics could be estimated from figures. Estimation was performed using WebPlotDigitizer (https://automeris.io/WebPlotDigitizer/). Estimated values were then used to calculate an effect size using one of the aforementioned steps. An overview audit of the method of derivation of each record effect size is provided as Supplementary Material as well as details of records that were suitable for inclusion but did not contain enough information to calculate an effect size. In those instances, study authors were contacted with a request to provide a correlation from their original data set, descriptive statistics, or points of clarification that would facilitate calculation of a correlation.

### Categories of Cognitive Function

**Figure 1.**
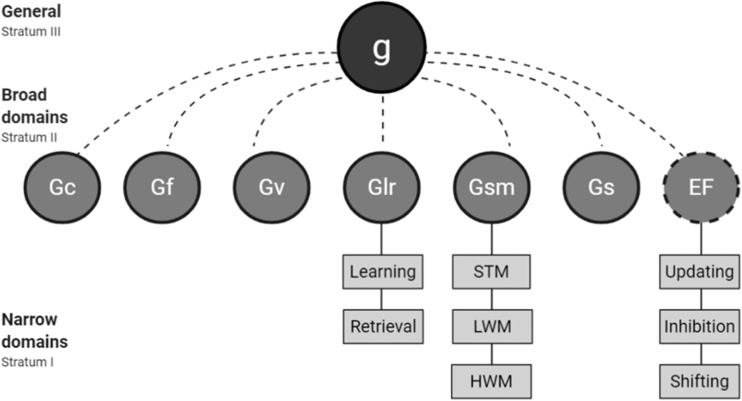
Hierarchical Representation of the CHC-M Composite Model (Adapted From Webb et al., 2018). g = general intelligence; Gf = fluid intelligence; Gc = crystallized intelligence; Gv = visual processing; Glr = long-term storage and retrieval; Gsm = general short-term memory; Gs = processing speed; EF = executive functions; STM = short-term memory; LWM = low-working memory; HWM = high-working memory. EF features a dotted outline to indicate its absence in the original CHC framework and subsequent addition by Webb et al. (2018) to create CHC-M.

The CHC-M cognitive taxonomy (Table 1) is based on a framework presented in Webb et al. (2018). CHC-M integrates both the Cattell–Horn–Carrol (CHC; Jewsbury et al., 2016) and Unity-Diversity (Friedman & Miyake, 2017) conceptualizations of executive function (EF). CHC theory originates from psychometric research, providing an extensively studied theoretical framework for conceptualizing human cognitive abilities (Carroll, 2010). The taxonomy presents a “three-stratum” taxonomical hierarchy of cognitive abilities. Each unique factor is identified as being distinct based on theoretically different processes and statistically divergent correlations (Webb et al., 2018). Atop the Atop the hierarchy (Figure 1) Stratum I is Charles Spearman’s general intelligence (g). Stratum II contains broad cognitive factors, including fluid intelligence (Gf), crystallized intelligence (Gc), visual processing (Gv), long-term storage and retrieval (Glr), general short-term memory (Gsm), and processing speed (Gs). Stratum III contains narrow cognitive factors that constitute Stratum II factors, for example, abstract reasoning and verbal reasoning constitute Gf. Although the original CHC model does not feature EF, the Unity-Diversity conceptualization of EF was incorporated by Webb et al. (2018). In this conceptualization, EF is comprised of the narrow factors of Updating, Inhibition, and Shifting. Unity-Diversity is considered synergistic with CHC, as it meets the same psychometric criteria applied in the original CHC model (i.e., theoretically different processes and statistically divergent correlations). The construct of executive functioning is of growing clinical interest, with related deficits being studied in various clinical populations such as depression, schizophrenia, and attention-deficit hyperactivity disorder.

**Table 1.** Descriptive Overview of the Broad and Narrow Cognitive Domains Constituting the CHC-M Taxonomy.

Broad cognitive domain		Narrow cognitive domain	
Fluid reasoning (Gf)	Deliberate but flexible control of attention to solve novel “on the spot” problems that cannot be performed by relying exclusively on previously learned habits, schemas, and scripts	Abstract reasoning	Ability to recognize (induct) and apply logical rules that govern sequence changes in abstract stimuli. Induction (I) and Sequential reasoning are defining features of Gf tasks
		Verbal reasoning	Ability to understand and evaluate the logic of various verbal arguments; reasoning with verbal material and knowledge acquired previously
Long-term memory and retrieval (Glr)	The ability to store, consolidate, and retrieve information over periods of time measured in minutes, hours, days, and years	Learning/encoding efficiency (Gl)	Ability for efficient learning of new information that is held over longer periods than what is typical of STM (Gsm) tasks
		Retrieval fluency (Gr)	The ability to rapidly access and recall information previously stored in long-term memory
General STM (Gsm)	The ability to encode, maintain, and manipulate information in one’s immediate awareness and includes memory span and working memory. The working memory literature distinguishes between single-task coordination (e.g., manipulation of one stream of stimuli in memory for retrieval) and multitask coordination (e.g., of two or more streams of information)	HWM	Multitask coordination: Manipulation/processing of multiple streams of information for a coordinated response. Can entail inhibition of one or more streamsEntails both processing and storage of multiple sources of information
		LWM	Entails a manipulation that is more cognitively complex than STM processes, but less complex than updating and HWM. Entails both processing and storage of a single (rather than multiple) source of information (e.g., recall a list in some specified order) other than how it was presented (e.g., in reverse order)
		STM	The ability to encode information, maintain it in immediate awareness (e.g., in primary memory), and immediately reproduce the information in the same sequence which it was presented
EF*	High-level cognitive processes, often associated with the frontal lobes that control lower level processes in the service of goal-directed behavior	Updating	The active process of monitoring incoming information and “updating” items held in working memory by replacing irrelevant information with task-relevant information
		Shifting	Shifting between mental sets or operations by disengaging from an irrelevant mental set and actively engaging in a set relevant to the current task
		Inhibition	Active and deliberate overriding of a dominant or automatic response in order to complete a task
Processing speed (Gs)	The ability to perform simple repetitive cognitive tasks quickly and fluently	Perceptual speed	The speed at which visual stimuli can be compared for similarity or difference
Visual processing (Gv)	The ability to make use of simulated mental imagery (often in conjunction with currently perceived images) to solve problems	Sensory perception	The efficiency of primary senses to process and provide information, picked up through primary senses (typically vision), necessary for task completion
		Visual perception	The ability to perceive complex patterns and mentally simulate how they might look when transformed (e.g., rotated, changed in size, partially obscured). It is central to Gv (like induction is central to Gf)

*Note.* Adapted from Webb et al. (2018). *EF definition from Friedman & Miyake (2017). EF = executive function; HWM = high-working memory; STM = short-term memory; LWM = low-working memory.

**Table 2.** Count of Effect Sizes in Each Broad Cognitive Factor.

Broad cognitive factors	Effect size total
EF	35
Gs	19
Glr	16
Cognitive screening	9
Gsm	8
Self-report	4
Gv	2

*Note.* EF = executive function; Gs = processing speed; Glr = long-term memory and retrieval; Gsm = general short-term memory; Gv = visual processing.

Supplementary Material from Webb et al. (2018) provides a categorization of cognitive tasks subsequently used to categorize cognitive tasks in this analysis. When a cognitive task did not feature in the Webb et al.’s (2018) categorization, two members of the review team independently categorized the task according the cognitive functions defined in the CHC-M taxonomy. Independent categorizations were compared, and consensus was achieved through discussion for instances where there was disagreement between categorizations.

In our published protocol (Clarke et al., 2018), we made the distinction between objective and subjective cognition (i.e., tasks that aim to measure a specific cognitive process vs. self-report of perceived cognitive performance). However, an unanticipated categorization issue arose in the form of clinical tests specifically designed to screen for mild cognitive impairment (MCI), such as the Montreal Cognitive Assessment (MoCA; Nasreddine et al., 2005). As MCI screening tests are designed to assess overall cognitive ability and not performance in specific cognitive domains, we categorized such tasks as “MCI screening tests.” A separate meta-analysis was conducted for this category.

### Statistical Analyses

All statistical analyses were performed in R (R Core Team, 2018). Meta-analyses were performed using the Metafor package (Viechtbauer, 2010). Random-effects models were fitted to data from broad cognitive domains (i.e., EF, Gs, Gsm, Glr, and Gv) and narrow cognitive domains where available data permitted. Due to the heterogeneous nature of records included in this analysis (i.e., studies produced from different laboratories, using different methods, and different populations), a random-effects model was chosen and specified prior to analysis (Clarke et al., 2018). As some models featured multiple effect sizes from a single study, the within-subjects statistical dependency of these effect sizes was accounted for using robust variance estimation (RVE; Hunter & Schmidt, 2004; Moeyaert et al., 2017; Quintana, 2015).

Model heterogeneity was evaluated using *Q* and *I*^2^ statistics. The *Q*-statistic tests the null hypothesis that all studies are evaluating the same effect. The *I*^2^ statistic ranges between 0% and 100% interpreted as to low (25%), moderate (50%), and high (75%) heterogeneity (Higgins et al., 2003; Quintana, 2015). Identification of potential outlying cases was performed in accordance with guidelines provided in Viechtbauer and Cheung (2010). Outlier diagnostics of the various meta-analytic models included: externally standardized residuals, difference of fits (DFFITS), Cook’s distances, covariance ratios, difference of beta weights (DFBETAS) values, estimates of *R*^2^ when each study is removed in turn, test statistics for (residual) heterogeneity when each study is removed in turn, diagonal elements of the hat matrix, and weights (in percentage) given to the observed outcomes during the model fitting. Baujat plots were used to visualize overly influential data points within models (Baujat et al., 2002; Quintana, 2015). A “leave-one-out” analysis was also performed on each model, which repeatedly fits the model, excluding a single study on each iteration, to verify that model results are not being driven by a single overly influential study.

Moderator analyses were performed through meta-regression models. Moderator variables were specified a priori and coded during the data extraction stage and included mean age of tinnitus participant sample, yes/no measurement of anxiety, and depression and hearing thresholds. Publication bias was assessed visually using contour-enhanced funnel plots. Statistical significance was tested using Rank correlation and Egger’s regression tests (Quintana, 2015). If a record could not be included within the meta-analyses, it was considered as part of the narrative synthesis.

## Results

### Overview

**Figure 2.**
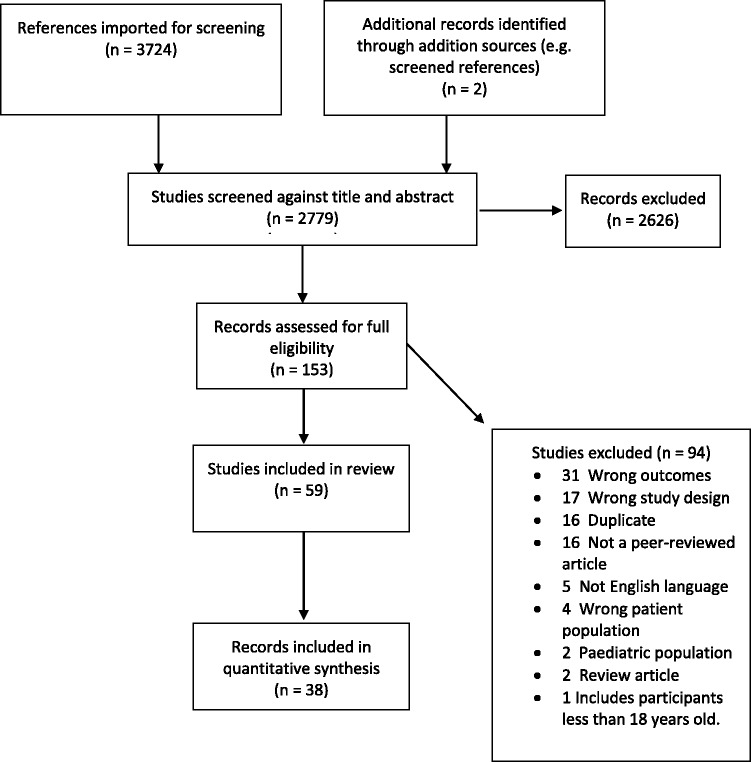
Search and Screening Overview Preferred Reporting Items for Systematic Review and Meta-Analysis Flowchart.

Thirty-eight records (1,863 participants) reported correlations or featured calculable correlations and were therefore included in the analyses (Figure 2). Ninety-three correlations were calculated from included records (Table 2).

### WoE—Record Bias and Quality Appraisal

The overall quality of studies varied widely. Assessment of the quality of each record in the context of its ability to answer the main review question was performed with WoE appraisal (Appendix). WoE scores were subsequently used as a variable within moderator analyses of the broad cognitive domain models.

### Model Results

An overview of the model results is provided in Table 3. The following sections detail analysis of each cognitive domain and the models that were applied.

**Table 3.** Overview of Meta-Analytic Models, Providing Correlation, Statistical Significance, and Number of Effect Sizes Contained in Its Calculation.

Model	*R*	*p*	Count
EF-rt	.35	<.001	24
EF-errors	.3	<.001	5
EF-correct	.1	*ns*	6
EF-Inhibition-rt	.32	<.001	14
EF-Inhibition-errors	.24	*ns*	2
EF-Shifting-rt	.48	<.001	8
EF-Shifting-errors	.37	.003	3
EF-Updating-rt	−.14	*ns*	2
Gs-rt	.34	<.001	13
Gs-errors	.27	.002	3
Gs-correct	−.2	*ns*	3
Gsm-correct	−.21	.024	7
Gsm-STM-correct	−.11	*ns*	3
Gsm-LWM-correct	−.31	*ns*	2
Gsm-HWM-correct	−.28	*ns*	2
Glr-correct	−.16	.003	16
Glr-GL-correct	−.14	.007	10
Glr-GR-correct	−.19	.024	6
Gv-rt	.3	*ns*	2
Self-report	.51	.04	4
Cognitive screening	−.47	.002	9

*Note.* EF = executive function; Gs = processing speed; Glr = long-term memory and retrieval; Gsm = general short-term memory; Gv = visual processing; HWM = high-working memory; STM = short-term memory; LWM = low-working memory; *ns* = not significant; rt = response time; GL = general learning, GR = general retrieval.

#### Executive Function

**Figure 3.**
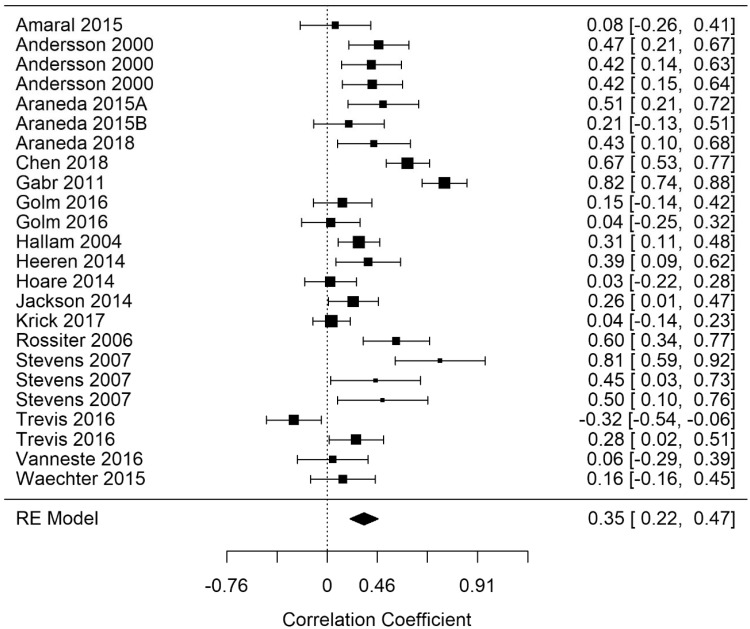
Forest Plot Showing the Correlation Between Tinnitus and Response Times in Executive Functioning Tasks (EF-rt). Each study is represented by a point estimate bounded by a 95% CI, with the area of each square proportional the study’s weight within the model. Summary effect size is displayed as a polygon at the bottom of the plot, with the width of the polygon representing the 95% CI. RE = Random-effects.

**Figure 4. fig4-2331216520918416:**
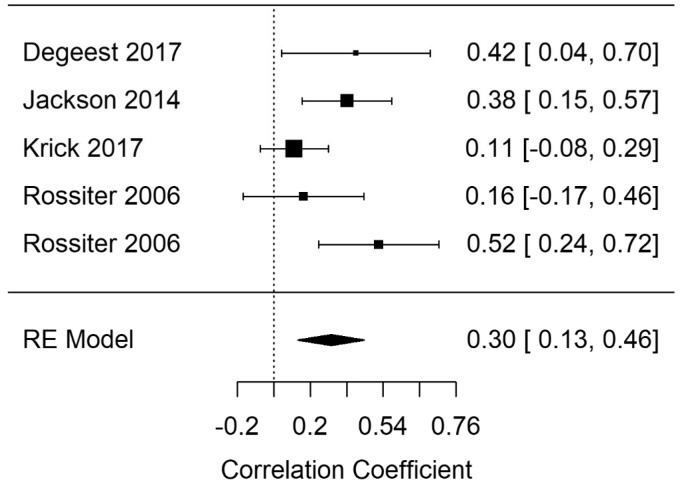
Forest Plot Showing the Correlation Between Tinnitus and Error Rates in Executive Functioning Tasks (EF-error). Each study is represented by a point estimate bounded by a 95% CI, with the area of each square proportional the study’s weight within the model. Summary effect size is displayed as a polygon at the bottom of the plot, with the width of the polygon representing the 95% CI. RE = Random-effects.

Random-effects meta-analyses for EF (broad domain) response time (EF-rt), errors (EF-errors), and correct responses (EF-correct) were performed. Figures 3 [Fig fig4-2331216520918416]to 5 show model summary correlations. Statistically significant summary correlations were identified in models for EF-rt (*r* = .35; 95% confidence interval [CI] [0.22, 0.47], *p* < .001) and EF-errors (*r* = .3; 95% CI [0.13, 0.46], *p* < .001). These results demonstrate longer response times and increased error rates in executive functioning tasks as tinnitus severity increased.

In a leave-one-out analysis, EF-rt remained statistically significant in every iteration, indicating that the result was not being driven by the effect of a single study. However, EF-errors was not statistically significant following the removal of Jackson et al. (2014), indicating that this study was solely responsible for driving the statistical significance of the original model. Analysis of EF-rt using RVE supported the statistically significant finding (r = .37; 95% CI [0.19, 0.54], *p* < .001).

The model for EF-rt featured a significant Q statistic, indicating that it did not share a common effect size Q(df =  23) = 143, p <.001). High and moderate heterogeneity was evident for both response time and error models. EF-rt (*I*^2^ = 81.98) and EF-error I_2_ = 51.2.

EF-correct was not statistically significant. Furthermore, as there were only six studies with the necessary outcome measures reported, there were too few data points to further investigate narrow EF domains with correct response measures.

Figure 6 shows a contour-enhanced funnel plot of EF-rt that suggests an absence publication bias. Egger’s regression and rank correlation tests were not significant, providing further support for a lack of publication bias within EF studies. Similarly, there was evidence for an absence of publication bias in all broad domain models, which are subsequently omitted for brevity.

Various moderator variables were tested in the EF-rt model to explain observed heterogeneity. These included presence/absence of hearing measurement, presence/absence of depression measurement, presence/absence of anxiety measurement, risk of bias quality score, and mean hearing threshold of tinnitus participants included in the record. No moderator variable was statistically significant within the broad EF domain. The number of broad EF studies available meant examination of EF narrow factors was also possible.

#### EF-Inhibition

Random-effects meta-analyses for Inhibition response time (Inhibition-rt) and errors (Inhibition-errors) were performed. A statistically significant summary correlation was identified for Inhibition-rt (*r* = .32; 95% CI [0.21, 0.43], *p* < .001). Figure 7 shows the Inhibition-rt summary correlation. Inhibition-error was not statistically significant. These results demonstrate longer response times for tasks measuring Inhibition as tinnitus severity increases. Results for this narrow factor include only three studies. RVE for Inhibition-rt supported the statistically significant finding with a significant point estimate (r = .34; 95% CI [0.16, 0.51], *p* = .002). Heterogeneity was reduced in the Inhibition-rt model, indicating that the cognitive subgrouping had reduced heterogeneity between studies from a large to a moderate amount (*I*^2^ = 58.94).

A Baujat plot and outlier diagnostics indicated that Stevens et al. (2007) may have been a potential outlier, having an overly influential effect on the overall result. A random-effects model was run with the removal of this study. The correlation remained statistically significant but was reduced from *r* = .32 to *r* = .28. However, removal of this study significantly reduced heterogeneity of the model from a large to moderate amount (*I*^2^ reduced from 58.94 to 37.86). Combined with a nonsignificant *Q* statistic, this may indicate that the remaining studies share a common effect size.

#### EF-Shifting

Random-effects meta-analyses for Shifting response time (Shifting-rt) and errors (Shifting-errors) were performed. A statistically significant summary correlation was identified for Shifting-rt (*r* = .48; 95% CI [0.24, 0.66], *p* < .001) and Shifting-errors (*r* = .37; 95% CI [0.13, 0.57], *p* = .003). Figure 8 shows the Shifting-rt summary correlation. RVE for Shifting-rt supported the statistically significant finding with a significant point estimate (r = .52; 95% CI [0.17, 0.86], *p* = .011). Response times and error rates for Shifting tasks increased as a function of tinnitus severity.

A Baujat and outlier diagnostics indicated that Gabr et al. (2011) may have been an outlier. The summary correlation remained statistically significant after the removal of Gabr et al. but with a smaller point estimate (*r* = .39; 95% CI [0.18, 0.57], *p* < .001). Although removal of this study significantly reduced heterogeneity of the model (*I*^2^ = reduced from 87 to 76.86), a large amount of heterogeneity remained across included studies.

#### EF-Updating

Only two studies included Updating effect sizes using response time outcomes (Updating-rt). The summary correlation for Updating-rt was not statistically significant.

#### Processing Speed (Gs)

Meta-analyses were performed on Gs response time (Gs-rt), errors (Gs-errors), and correct (Gs-correct). Figure 9 shows the Gs-rt summary correlation. Statistically significant summary correlations were identified in models for Gs-rt (*r* = .34; 95% CI [0.17, 0.49], *p* < .001) and Gs-errors (*r* = .27; 95% CI [0.1, 0.43], *p* = .002). These results demonstrate longer response times and increased error rates in processing speed tasks as tinnitus severity increased.

The summary correlation for Gs-correct was not statistically significant. RVE for Gs-rt supported the statistically significant finding with a significant point estimate (r = .35; 95% CI [0.14, 0.56], *p* = .004). RVE could not be performed for Gs-errors due to lack of available degrees of freedom. A leave-one-out analysis for Gs-rt, which remained statistically significant in every iteration, indicating that the result was not being driven by the effect of a single study.

A Baujat plot indicated that Gabr et al. (2011) may be overly influential on the Gs-rt model. Gs-rt was rerun with the removal of this study. The summary correlation remained statistically significant but with a smaller point estimate (*r *= .28; 95% CI [0.14, 0.42], *p* < .001). RVE for Gs-rt supported the statistically significant finding with a significant point estimate (r = .29; 95% CI [0.12, 0.46], *p* = .005). Although removal of this study significantly reduced heterogeneity of the model (*I*^2^ reduced from 81.6 to 69.72), moderate heterogeneity remained across included studies.

Various moderator variables were tested in the Gs-rt model to explain observed heterogeneity. These included presence/absence of hearing measurement, presence/absence of depression measurement, presence/absence of anxiety measurement, risk of bias quality score, and mean hearing threshold of tinnitus participants included in the record. The model including presence/absence of depression measurement produced a statically significant moderator coefficient (*p* = .037). This suggests that a proportion of the slower response times observed on processing speed tasks were confounded by inclusion of participants with depression, with *I*^2^ being reduced from 81.63 to 75.12.

#### General Short-Term Memory (Gsm)

Gudwani et al. (2017) reported errors as an outcome measure; it was therefore excluded from the analysis as the remaining studies reported number of correct responses. A random-effects meta-analysis was performed on the remaining studies (Gsm-correct). Figure 10 shows the Gsm-correct summary correlation. A statistically significant summary correlation was calculated for Gsm-correct (*r* = −.21; 95% CI [−0.38, −0.03], *p* = .024). The results demonstrate less correct answers on general short-term memory tasks as tinnitus severity increased. RVE supported the finding with a significant point estimate (−.22; 95% CI [−0.43, −0.001], *p* = .049). Moderate heterogeneity was present in the Gsm-correct model (*I*^2^ = 72.67).

In a leave-one-out analysis, statistical significance was not reached in two iterations, indicating that the result was being driven by the effects of two influential studies. Regression diagnostics indicated no evidence of outliers in Gsm-correct.

A model including presence/absence of hearing measurement as a moderating variable produced a statistically significant moderator coefficient (*p* = .047). This suggests that the fewer correct responses observed in general short-term memory tasks were confounded by inclusion of participants with hearing loss. *I*^2^ in the models with hearing impairment reduced from 72.69 to 62.71.

#### Short-Term Memory, Low-Working Memory, and High-Working Memory

Random-effects meta-analyses were conducted on Gsm narrow factors for short-term memory (Gsm-STM), low working memory (Gsm-LWM), and high working memory (Gsm-HWM). None of the narrow factor models were statistically significant.

#### General Learning and Retrieval (Glr)

A random-effects meta-analysis was performed for Glr (Glr-correct). Figure 11 shows the Glr-correct summary correlation. A statistically significant summary correlation was calculated for Glr-correct (*r* = −.16; 95% CI [−0.24, −0.07], *p* < .001). The results demonstrate less correct answers on general learning and retrieval tasks as tinnitus severity increased. RVE supported the finding with a significant point estimate (−.16; 95% CI [−0.31, −0.01], *p* = .04). Moderate heterogeneity was present in the Glr-correct model (*I*^2^ = 41.31). A leave-one-out analysis for Glr-correct remained statistically significant in every iteration, indicating that the result was not driven by the effect of a single study.

Moderator analysis for Glr-correct found that no variable accounted for a statistically significant amount of heterogeneity. Random-effects meta-analyses were ran on Glr narrow factors for learning (Glr-learning) and retrieval (Glr-retrieval).

#### General Learning (Gl)

Figure 12 shows the Glr-GL-correct summary correlation. The summary correlation for Glr-learning was statistically significant (*r* = −.14; 95% CI [−0.25, −0.04], *p* = .03). The results demonstrate less correct answers when performing memory retrieval tasks as tinnitus severity increased. Moderate heterogeneity was present in the Glr-learning model (*I*^2^ = 29.2).

#### General Retrieval (Gr)

Figure 13 shows the Glr-GR-correct summary correlation. The summary correlation for Glr-retrieval was statistically significant (*r* = −.19; 95% CI [−0.34, −0.03], *p* = .024). The results demonstrate less correct answers when performing memory retrieval tasks as tinnitus severity increases. Moderate heterogeneity was present in the Glr-retrieval model (*I*^2^ = 59.84).

#### Visual Processing (Gv)

A random-effects meta-analysis was performed for the Gv (Gv-rt). The summary correlation for Gv-rt was not statistically significant.

#### Cognitive Self-Report

Figure 14 shows the self-report summary correlation. The summary correlation for cognitive self-report was statistically significant (*r* = .51; 95% CI [0.03, 0.8], *p* = .04). The results demonstrate poorer self-reported cognition as tinnitus severity increased. High heterogeneity was present in the cognitive self-report model (*I*^2^ = 92.73). A leave-one-out analysis showed that the observed effect was likely driven by a single study. The effect size without Alam et al. (2012) showed a smaller but still statistically significant point estimate (*r* = .28; 95% CI [0.14, 0.41], *p* < .001). Moderator analysis showed that no single variable was significant for any cognitive self-report model.

#### Cognitive Impairment Screening

Figure 15 shows the MCI summary correlation. The summary correlation for MCI was statistically significant (*r* = −.47; 95% CI [−0.68, −0.18], *p* = .002). The results show poorer performance on cognitive screening tasks for participants with tinnitus. RVE supported the finding, with a statistically significant point estimate (−.5; 95% CI [−0.97, −0.04], *p* = .04). High heterogeneity was present in the cognitive screening model (*I*^2^ = 93.42). In a leave-one-out analysis of cognitive impairment screening, all models remain statistically significant, that is, the effect was not driven by a single study.

## Discussion: Is Tinnitus Associated With Poorer Cognitive Performance?

This review provides a profile of cognitive performance associated with tinnitus through different types of outcome measures used in tasks measuring cognitive performance (i.e., response time, accuracy, and error rates) across various cognitive domains. Having tinnitus is associated with longer responses times and increased error rates in processing speed (Gs) and EF tasks and less correct answers on general short-term memory (Gsm) and general learning and retrieval (Glr) tasks. Being able to incorporate the different outcome measures used across the varying studies meant that this review was comprehensive in its scope, providing a broad synthesis of the evidence of tasks that have investigated any aspect of tinnitus and cognitive performance. In doing so, the analysis illuminates a general trend of significantly poorer performance in participants with tinnitus as well as estimating the relative size of this effect within each cognitive domain. The pattern of associations outlined in this analysis provides a framework for theorizing about various cognitive domains and their relationship to tinnitus. This is important as current evidence suggests that cognitive training, particularly of EFs, may improve aspects of cognition (Diamond & Ling, 2016); this could serve to remediate poorer cognitive performance, but its impact on perceived severity of the tinnitus is unknown. The following sections consider associations between tinnitus and specific broad and narrow cognitive factors within the CHC-M taxonomy (i.e., Strata I and II).

### Which Broad Cognitive Domains Are Associated With Tinnitus?

Subjective tinnitus is associated with poorer performance in tasks measuring EF, general short-term memory, long-term storage and retrieval, and processing speed. There were no studies that featured tasks measuring the broad cognitive factors of fluid intelligence (Gf) and crystallized intelligence (Gc).

#### Executive Function

This review demonstrates consistently poorer EF performance by individuals with tinnitus. This is evidenced through increased response times and errors rates in EF-rt and EF-error models. No significant moderating variables were identified within the domain of executive functioning. The models applied within this synthesis likely reflect high heterogeneity that is typical within the construct of executive functioning, given their proposed role in providing cognitive control and coordination of “lower level” cognitive systems (Miyake et al., 2000). Subsequently, EF has been a theoretically challenging construct to research for various methodological reasons (cf., Rabbitt, 2004 for a review). Despite this, recent theorizing has used psychometric techniques such as factor analysis, which have facilitated consensus regarding three “core” EFs: Updating, Inhibition, and Shifting (Diamond, 2013; Miyake & Friedman, 2012). Associations between tinnitus and individual core EFs are considered later when discussing narrow cognitive domains.

#### Processing Speed (Gs)

Increased errors rates and response times in processing speed tasks were demonstrated through the Gs-rt and Gs-error models. The moderate correlation remained significant despite the removal of a potential outlier. Analysis of moderator variables revealed a significant effect of whether depression was measured as a confounding variable within the study. This suggests that some of the observed effect could be confounded by inclusion of participants with depression, which is known to be associated with impaired cognitive performance (Rock et al., 2014). Due to depression having an established association with severe tinnitus (Bhatt et al., 2017), it is necessary to consider it as a potentially confounding factor in the demonstrated association between tinnitus and cognitive performance. Adequate measurement of depression is therefore important to consider as a statistical “control” within analyses in primary studies.

Interestingly, summary correlations for both processing speed and EF response time tasks were similar in size. This may be due to EFs being required to perform even seemingly simple tasks. Processing speed is thought to represent the speed with which an individual processes information, and tasks described as measuring processing speed are typically claimed to reflect underlying neural speed, efficiency, and capacity. While slower processing speed is associated with tinnitus, authors have noted that processing speed tasks are seldom free of cognitive control and therefore the association found between tinnitus and processing speed may reflect an artifact of an association between tinnitus and EF (Cepeda et al., 2013; Duncan et al., 2005).

#### General Short-Term Memory (Gsm)

The Gsm-correct model demonstrated poorer performance on general short-term memory tasks for participants who have tinnitus. However, there were only seven effect sizes used in this analysis. Furthermore, a leave-one-out analysis indicated that the statistically significant finding was not as reliable as the findings of EF analyses and was driven by two studies.

A moderator model that included whether hearing thresholds were measured was statistically significant and explained over 30% more variance. Hearing impairment associated with tinnitus and a known potential confounding factor for cognitive performance due to a known association with poorer overall cognition (Dawes et al., 2015). It is therefore difficult to arrive at firm conclusions with the available data concerning the association between tinnitus and short-term memory function. Previous reviews have reported mixed findings and an inability to form conclusions based on the available evidence. They attribute this to variability in tasks and controls featured within studies (Mohamad et al., 2016; Trevis et al., 2018). Our review builds on this conclusion by demonstrating that hearing impairment is likely to be a confounding factor in the available evidence.

#### General Learning and Retrieval (Glr)

The Glr-correct model demonstrated an association between tinnitus and performance in general learning and retrieval tasks. This is an interesting finding as several theories have suggested that long-term memory retrieval could be a significant feature of tinnitus generation and maintenance (De Ridder et al., 2014; Sedley, 2019). Previous theoretical reviews of tinnitus generation have posited a potentially important role for memory; however, this has typically focused on short-term and working-memory capacity. This analysis demonstrates an association between tinnitus and long-term memory retrieval. Furthermore, we were able to analyze specific narrow cognitive factors of general learning/encoding (Gl) and general retrieval (Gr), which are both considered later.

### Which Narrow Cognitive Domains Are Associated With Tinnitus?

Tinnitus is associated with EF-Inhibition and EF-Shifting narrow factors within executive functioning (i.e., within Stratum I). More data are required to form conclusions regarding EF-Updating. While general short-term memory was found to be significantly associated with tinnitus, none of its constituent narrow factors were significant in isolation. This is likely due to insufficient data or the task choice not being specific to the theoretical aspect of general short-term memory under investigation. EF-Updating represents a promising cognitive factor for further research. Both general learning and general retrieval were found to be significantly associated with tinnitus. This cognitive domain is of emerging interest in cognitive performance research related to tinnitus.

#### EF-Inhibition, EF-Shifting, and EF-Updating

While no significant correlation was calculated for the EF-Updating factor, only two studies were available to contribute data. Due to the significant association with other EF constructs, EF-Updating remains an important theoretical construct to consider (Ecker et al., 2010, 2014). For example, Trevis et al. (2016) used the *N*-back task and found statistically significant poorer performance between a group with subjective tinnitus compared with a control group. This suggests that all “core” EFs are associated with tinnitus. Updating is intricately related to general short-term memory through being a theoretical functional component of working memory. Although several studies have included working-memory measures, they have used capacity measures, which attempt to measure limitations of short-term memory storage while performing operations, not the ability to update or refresh short-term memory.

#### Short-Term Memory, Low-Working Memory, and High-Working Memory

While the broad general short-term memory factor showed significantly poorer performance in participants with tinnitus, analysis of the narrow factors found that none were significant in isolation. This could be due to few available data points within each model; however, it may also result from the use of capacity tasks in previous study designs. These tasks may be insensitive measures of the Updating aspect of working memory, which can be conceptualized as a “refreshing” of a memory buffer and which may be less efficient in people with tinnitus. Poorer performance in narrow executive functioning factors of Inhibition and Shifting is suggestive that tasks that operationalize Updating may be more specific in measuring working-memory deficits in people with tinnitus. This has empirical support from a study by Trevis et al. (2016) that used an *N*-back task, which better conceptually represents Updating; the authors reported a significant difference on performance on this task between participants with and without tinnitus. Webb et al. (2018) note that the literature distinguishes between two types of working-memory task: those that deal with one stream of stimuli (for retrieval) and those that deal with multi-task coordination (i.e., two or more streams of information). It is interesting to note that many tasks analyzed in this review were capacity tasks (Schmiedek et al., 2009).

#### General Learning (Gl) and General Retrieval (Gr)

Analysis of these narrow factors demonstrated a significant association between tinnitus and both general learning (Gl) and general retrieval (Gr). Participants with tinnitus performed worse on tasks of long-term storage and retrieval of information from long-term memory. General learning and retrieval tasks have typically featured as control tasks in studies that were primarily interested in other domains of cognitive performance linked to tinnitus. The different sized summary correlations and CIs of general learning and general retrieval indicate that poorer general retrieval performance is more likely (i.e., narrow CI) than general learning. It is also important to note that tasks investigating the ability to perform “rapid retrieval” (i.e., pressured retrieval within a small timeframe) will necessarily involve executing strategy via executive functioning, which may moderate the observed association.

### Cognitive Self-Report

Previous reviews have suggested more self-reported cognitive failures for participants who have tinnitus. This analysis revealed a significant association between tinnitus and self-reported cognitive performance; however, this is driven by the large effect reported in Alam et al. (2012). According to the results of this analysis, the association between tinnitus severity and perception of cognitive performance is likely to be more modest. All studies in this analysis used the Cognitive Failures Questionnaire (Broadbent et al., 1982). Self-reported cognitive difficulties may be better assessed in a clinical tinnitus population through a specific screening tool such as the cognitive scale of the Tinnitus Functional Index.

### Cognitive Impairment Screening

Both objective and subjective measures of cognitive performance were considered in this review as per our published protocol (Clarke et al., 2018). However, during the review process, it became evident that a distinct category was required for tests that screen for cognitive impairment. Such paper-and-pencil tests include the MoCA, Mini State Mental Examination (MMSE), and the Cognitive Abilities Screening Questionnaire (Devenney & Hodges, 2017; Nasreddine et al., 2005; Teng et al., 1994). Such measures are typically used in a clinical setting to screen for cognitive impairment in diseases such as dementia. It is important to note that the moderate correlation demonstrated with this analysis is being driven by two studies that feature reported correlations (i.e., correlations reported in the study manuscript, not calculated by the review team), which are unusually large (Lee et al., 2020; Wang et al., 2018). The correlation reported by Lee et al. (2020) is unusually large, and the reported association is likely confounded by having an older mean sample of tinnitus participants. While some studies have suggested that cognitive screening tools have clinical utility (Lee et al., 2020), it is uncertain how sensitive they are for patients with cognitive problems associated with tinnitus in addition to MCI per se. Consideration may therefore be given to using both a cognitive impairment screening tool and a specific tinnitus related concentration measure. Furthermore, choice of cognitive screening instrument is an important consideration, as with tasks of cognitive performance. In this review, there was large association between tinnitus and the MoCA cognitive screening tool. This provides some limited support for the main study findings of an association between tinnitus and EF because the MoCA includes short tasks that test EFs and was designed to investigate cognitive processes not tapped by the MMSE (Ciesielska et al., 2016).

## Quality of Available Evidence

Although this analysis has demonstrated associations between tinnitus and several domains of cognition, there are methodological and conceptual considerations inherent in the available data that preclude a conclusion of tinnitus per se causing poorer memory or cognitive control, as has been suggested in previous studies (Heeren et al., 2014; Pierce et al., 2012; Schultz et al., 2018; Tegg-Quinn et al., 2016). Understanding the quality of the evidence currently available concerning associations between tinnitus and cognitive performance is important for developing models of tinnitus generation, its maintenance, and its perceived severity; this understanding will facilitate the identification of targets for intervening on elements within prospective models. For example, both cognitive training (broadly construed) and EF training specifically are potential interventions for treating tinnitus (Diamond & Ling, 2016; Krings et al., 2015). This analysis initially appears to suggest that cognitive factors such as processing speed, short-term memory, long-term memory retrieval, and executive functioning are all appropriate targets for a training intervention; however, the quality of the available evidence suggests that EF training is a more promising interventional target due to its larger associations with tinnitus, while processing speed and short-term memory domains feature potential confounding through depression and age. Furthermore, with an increased research interest in cognitive aspects of tinnitus being displayed in recent years, it is crucial that researchers are aware of various methodological and confounding factors that will affect conclusions that are drawn from primary research. The following sections discuss the pervasive issue of confounding, methodological issues within currently available evidence, and specific limitations to the conclusions that can be reached as a result of this synthesis.

### Confounding Variables

Although these analyses did not find age to be a statistically significant moderator within any model, this is likely due to the unit of analysis within models being studies not individuals, which may mask the effects of age on cognitive performance at the within-study level. Many studies within this review featured similarly aged samples, with approximately 70% of the meta-analyzed records reporting a mean sample age of 40 to 50 years. It is well established that absolute response time when performing cognitive tasks increases as a function of age so much so that the utility of a null model within the cognitive gerontological literature has been dubbed the “dull hypothesis” (Perfect & Maylor, 2000; Salthouse, 1996; Verhaeghen, 2013). Future research should therefore ensure age is accounted for by matching groups or including age as a covariate in data analyses.

Participant hearing acuity is a further potential confounding factor requiring appropriate consideration in future research, with current evidence demonstrating associations between poorer cognition and reduced hearing thresholds (Wayne & Johnsrude, 2015). Reduced hearing thresholds are also associated with an increased prevalence of tinnitus. Although 31 studies within the review measured hearing using pure tone audiometry, the range of test frequencies measured, and subsequent reporting of mean average hearing thresholds were inconsistent, precluding detailed coding of this variable. To facilitate the future syntheses and comparisons between studies, it is important to use a uniform criteria such as those provided by the World Health Organization or British Society of Audiology (2018).

Prevalence of depression and anxiety symptoms increases with tinnitus severity (Bhatt et al., 2017) and both are known to be associated with cognitive performance. Twenty-seven and 24 studies, respectively, included measurements of depression and anxiety. Sparseness of studies that did so within each cognitive domain, combined with differences in the questionnaires that were used (e.g., state or trait measures), made it difficult to synthesize the studies and understand their potential confounding influence. Future research would benefit from considering adequate measurement of depression and anxiety or featuring clinical levels as an exclusion criteria to exclude the contribution of these factors.

### Methodological Considerations

Few studies within this review report an *a priori* power calculation. A lack of statistical power limits the conclusions that can be drawn from published research. This synthesis makes clear that accurate estimates of associations between tinnitus and cognitive performance require adequately powered studies. This is particularly relevant in psychological research given recent debate concerning a “replicability crisis” (Open Science Collaboration, 2015). Future research investigating links between tinnitus and cognitive performance would benefit from consideration of how best to obtain adequate inferential statistical power, such as sufficient sample size, and could power studies based on findings within this meta-analysis. For example, given that the summary correlations we typically observed are of the order of .3 or less, at least 85 participants would be required for 80% power at a significance level of .05 (Cohen, 1988, p. 100, Table 3.4.1). Further to the issue of sufficiently powering studies through appropriate sample size, we also note that approximately one in three studies within the reviewed literature failed to report the severity of tinnitus in sampled participants, while the quality with which tinnitus was defined within-study inclusion/exclusion criteria was also notably variable.

A crucial methodological consideration for cognitive performance studies is the specific cognitive task used. Mohamad et al. (2016) suggested that multiple tasks should be used to assess the cognitive domain of interest. They also noted the importance of using validated tasks. Due to task impurity, “validation” in a cognitive sense is tantamount to majority consensus within the literature. It is therefore important to consider the underlying theory when investigating any potential behavioral impact of tinnitus using cognitive tasks. For example, the go/no-go task paradigm has been used as a measure of EF-Inhibition, but Diamond (2013) notes that this appears to tap a special case of Inhibition, with the construct being arguably better operationalized with the color-word Stroop paradigm. Theoretical specificity of tasks assessing working memory and its constituent components should also be given due to consideration in future work, such as complex span tasks versus tasks of Updating (Ecker et al., 2010; Schmiedek et al., 2009). A major area of study under such models has been trying to attain understand the “capacity” of working memory, with many tasks that are used as working-memory measures being capacity measures when Updating measures may be more appropriate in a tinnitus population (Oberauer, 2019; Schmiedek et al., 2009; Wilhelm et al., 2013).

Given the findings of this review, cognitive tasks that tap fluid ability (Gf) may be of interest in future tinnitus research. Crystallized intelligence (Gc) is the sum of knowledge acquired through the lifespan, and as such typically increases over the course of a person’s life, while Gf encompasses the ability to solve novel problems (McGrew, 2009). Diamond (2013) suggests that Gf is essentially the joint functioning of core EFs (Updating, Inhibition, and Shifting). Therefore, Gf measures may be of particular interest in assessing clinical tinnitus populations; for example, although the MMSE has been the more popular MCI screening measure, the MoCA taps executive functioning and may therefore be more sensitive to concentration difficulties accompanying bothersome tinnitus.

### Synthesis Strengths and Limitations

A primary strength of this review is its bias limiting methods. The first was registration of the protocol on PROSPERO database (CRD42018085528). Second, a detailed protocol of the planned analysis was published in a peer-reviewed journal in advance of the review being completed (Clarke et al., 2018). Third, we limited bias produced through excessive researcher degrees of freedom through task impurity and subsequent categorization of cognitive tasks, which may enable authors to pool studies in a manner that would produce a statistically significant finding, justified in a post hoc manner. Using an existing cognitive taxonomy removed the pooling decision from the review team, limiting opportunities to introduce author bias. In addition to reducing author bias, CHC-M taxonomy provides a cogent, theoretically derived analytic framework by which to structure a broad analysis of associations between tinnitus and cognitive performance. It also provided a theoretically driven means of focusing meta-analyses to obtain maximum value from existing data available within the literature.

A limitation of this review is the varying amount of data available across the various broad cognitive factors (Table 2).” It is clear from the counts of observations across broad cognitive categories that the majority of research to date concerning tinnitus and cognition has focused on attention (Acrani & Pereira, 2010; Cuny et al., 2004; Heeren et al., 2014; Husain et al., 2015; Mohamad et al., 2016; Roberts et al., 2013; Rossiter et al., 2006; Searchfield et al., 2007; Spiegel et al., 2015; Tegg-Quinn et al., 2016). This is evident as we were able to calculate 3 times as many effect sizes for executive functioning compared with processing speed (the cognitive category with the second largest number of observations).

High heterogeneity was evident across studies in many of the models in this review. This is unsurprising as many different kinds of studies were included. There were many anticipated sources of nonrandom variation, such as how tinnitus was defined and measured, hearing thresholds of participants, measurement of depression, or anxiety. Our analyses anticipated sources of variation through a priori specification of data items to extract as moderating variables (Clarke et al., 2018), but few of these were found to be statistically significant within the models tested. This suggests that other factors accounted for the high heterogeneity noted in many models. We suggest that a major contributor to the unexplained variance may be the type of task that was used in each study. While included studies cover many of the broad cognitive domains, some tasks operationalize these theoretical domains better than others. For instance, some tasks such as Test of Everyday Attention have been used in good quality studies but failed to observe findings of poorer performance in participants with tinnitus (Hoare et al., 2014). While such tasks have high face validity of measuring “attention,” they are unlikely to tap a specific theoretical factor within executive functioning, but instead put minimal load on several narrow factors.

## Conclusions

The available data indicate that tinnitus is associated with poorer performance across a variety of broad cognitive domains including executive functioning (EF), processing speed (Gs), general short-term memory (Gsm), and general learning and retrieval (Glr). This analysis also indicates that specific narrow cognitive domains are associated with tinnitus to varying extents; these include Inhibition and Shifting (within EF) and learning and retrieval (within Glr). However, findings should be considered in light of the quality of the available evidence. Future research concerning associations between tinnitus and cognitive performance should appropriately consider the conceptual and methodological issues observed in the present evidence, such as statistical power and confounding factors.

**Figure 5.**
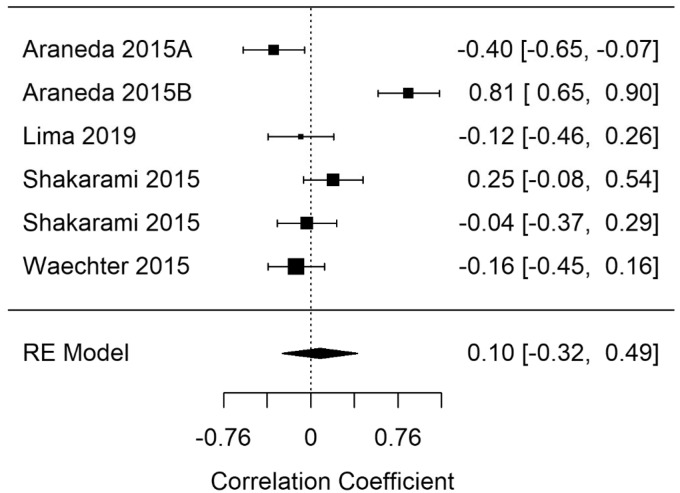
Forest Plot Showing the Correlation Between Tinnitus and Correct Responses in Executive Functioning Tasks (EF-correct). Each study is represented by a point estimate bounded by a 95% CI, with the area of each square proportional the study’s weight within the model. Summary effect size is displayed as a polygon at the bottom of the plot, with the width of the polygon representing the 95% CI. RE = Random-effects.

**Figure 6.**
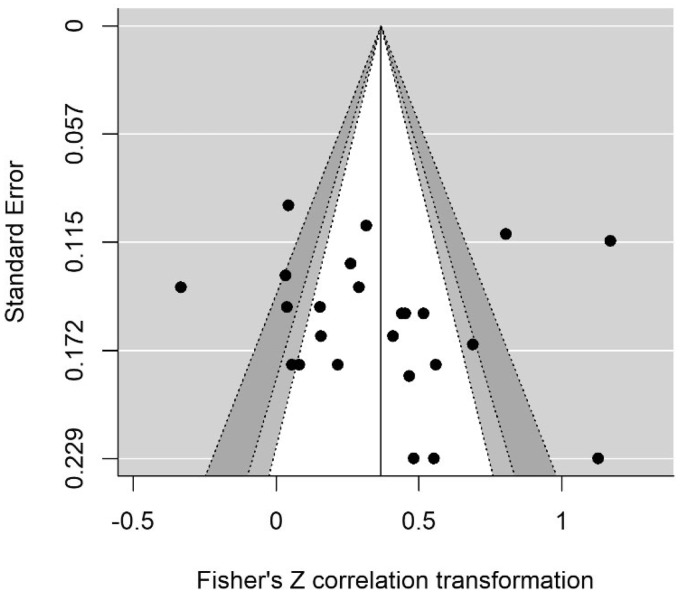
Contour-Enhanced Funnel Plot for EF-rt Model. Individual study effect sizes are displayed on the *x*-axis, with their corresponding standard errors on the *y*-axis. The funnel lines are centered on the summary effect size, providing an indication of the spread. The unshaded region in the middle corresponds to *p* values greater than .10, the light gray shaded region corresponds to *p* values between .10 and .05, the dark gray region corresponds to *p* values between .05 and .01, and the region outside of the funnel corresponds to *p* values below .01.

**Figure 7.**
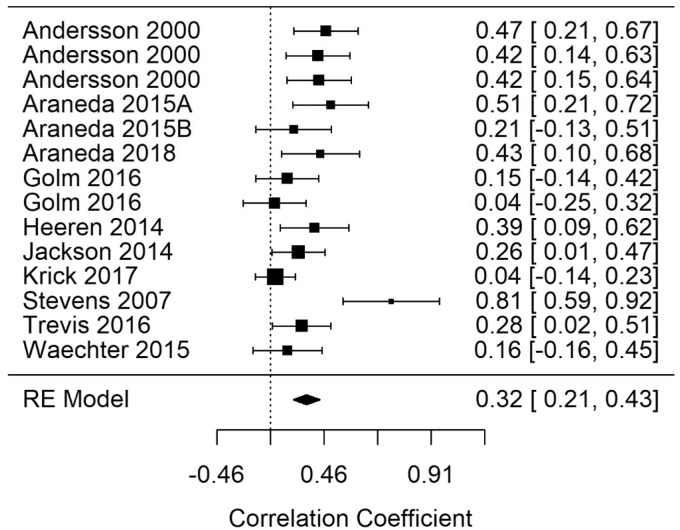
Forest Plot Showing the Correlation Between Tinnitus and Response Times in Inhibition Tasks (Inhibition-rt). Each study is represented by a point estimate bounded by a 95% CI, with the area of each square proportional the study’s weight within the model. Summary effect size is displayed as a polygon at the bottom of the plot, with the width of the polygon representing the 95% CI. RE = Random-effects.

**Figure 8.**
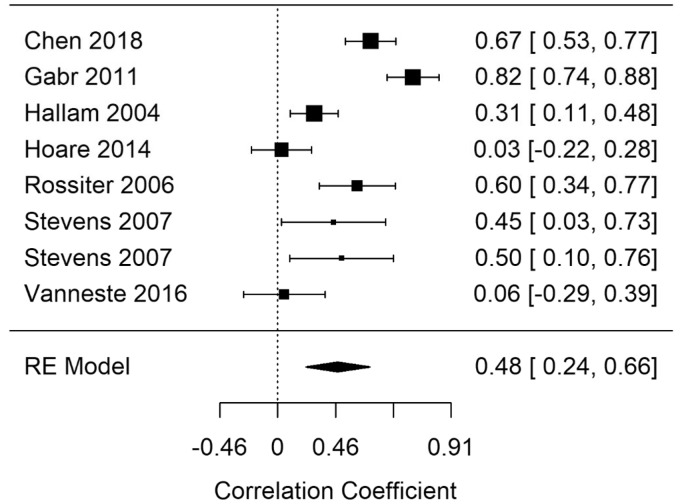
Forest Plot Showing the Correlation Between Tinnitus and Response Times in Shifting Tasks (Shifting-rt). Each study is represented by a point estimate bounded by a 95% CI, with the area of each square proportional the study’s weight within the model. Summary effect size is displayed as a polygon at the bottom of the plot, with the width of the polygon representing the 95% CI. RE = Random-effects.

**Figure 9.**
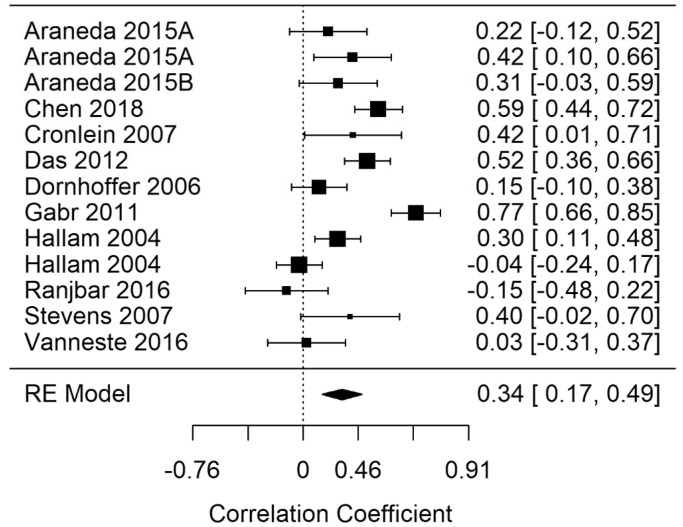
Forest Plot Showing the Correlation Between Tinnitus and Response Times in Processing Speed Tasks (Gs-rt). Each study is represented by a point estimate bounded by a 95% CI, with the area of each square proportional the study’s weight within the model. Summary effect size is displayed as a polygon at the bottom of the plot, with the width of the polygon representing the 95% CI. RE = Random-effects.

**Figure 10.**
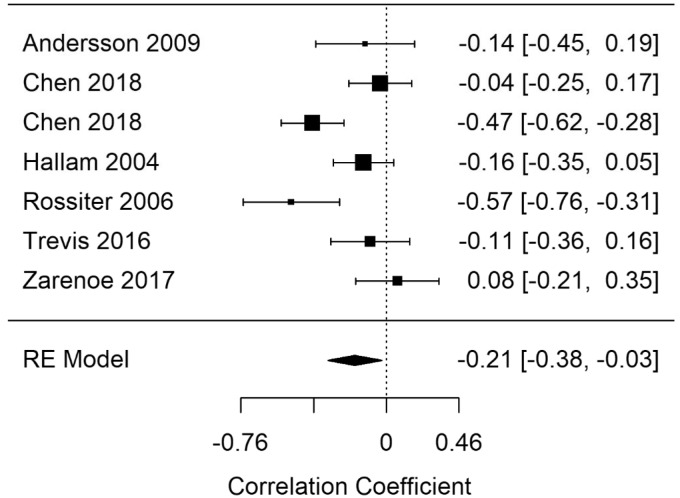
Forest Plot Showing the Correlation Between Tinnitus and Correct Responses in General Short-Term Memory Tasks (Gsm-correct). Each study is represented by a point estimate bounded by a 95% CI, with the area of each square proportional the study’s weight within the model. Summary effect size is displayed as a polygon at the bottom of the plot, with the width of the polygon representing the 95% CI. RE = Random-effects.

**Figure 11.**
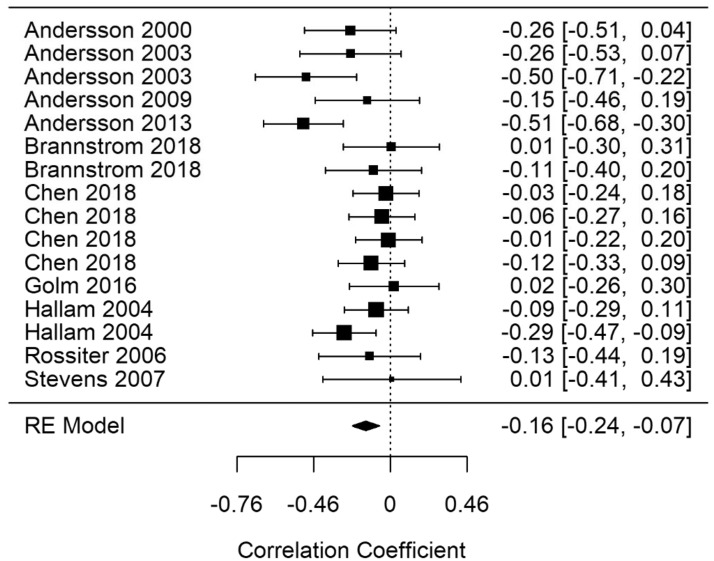
Forest Plot Showing the Correlation Between Tinnitus and Correct Responses in General Learning and Retrieval Tasks (Glr-correct). Each study is represented by a point estimate bounded by a 95% CI, with the area of each square proportional the study’s weight within the model. Summary effect size is displayed as a polygon at the bottom of the plot, with the width of the polygon representing the 95% CI. RE = Random-effects.

**Figure 12.**
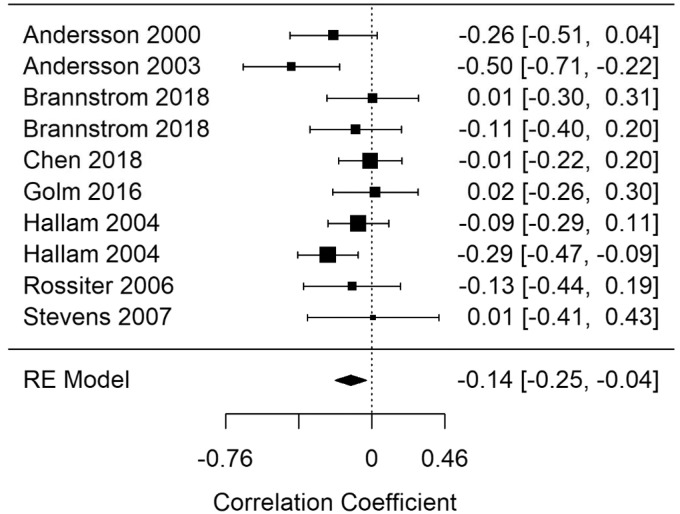
Forest Plot Showing the Correlation Between Tinnitus and Correct Responses in General Learning Tasks (Glr-GL-correct). Each study is represented by a point estimate bounded by a 95% CI, with the area of each square proportional the study’s weight within the model. Summary effect size is displayed as a polygon at the bottom of the plot, with the width of the polygon representing the 95% CI. RE = Random-effects.

**Figure 13.**
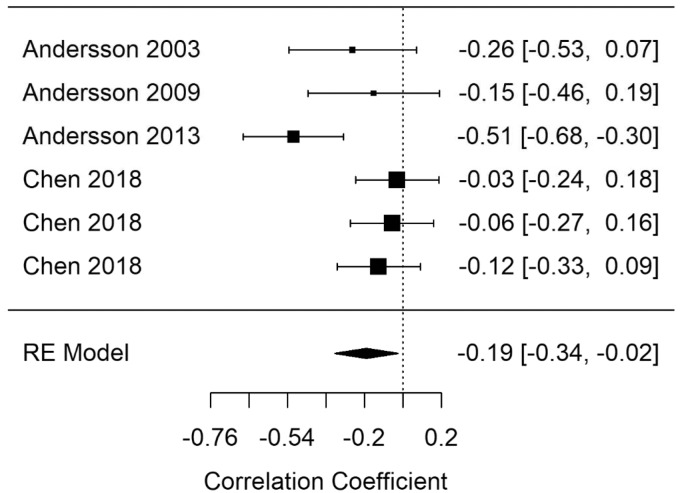
Forest Plot Showing the Correlation Between Tinnitus and Correct Responses in General Retrieval Tasks (Glr-GR-correct). Each study is represented by a point estimate bounded by a 95% CI, with the area of each square proportional the study’s weight within the model. Summary effect size is displayed as a polygon at the bottom of the plot, with the width of the polygon representing the 95% CI. RE = Random-effects.

**Figure 14.**
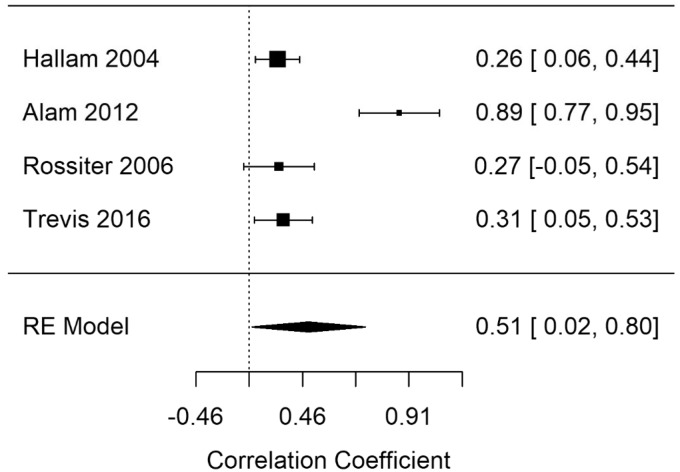
Forest Plot Showing the Correlation Between Tinnitus and Cognitive Self-Report Measures (Self-report). Each study is represented by a point estimate bounded by a 95% CI, with the area of each square proportional the study’s weight within the model. Summary effect size is displayed as a polygon at the bottom of the plot, with the width of the polygon representing the 95% CI. RE = Random-effects.

**Figure 15.**
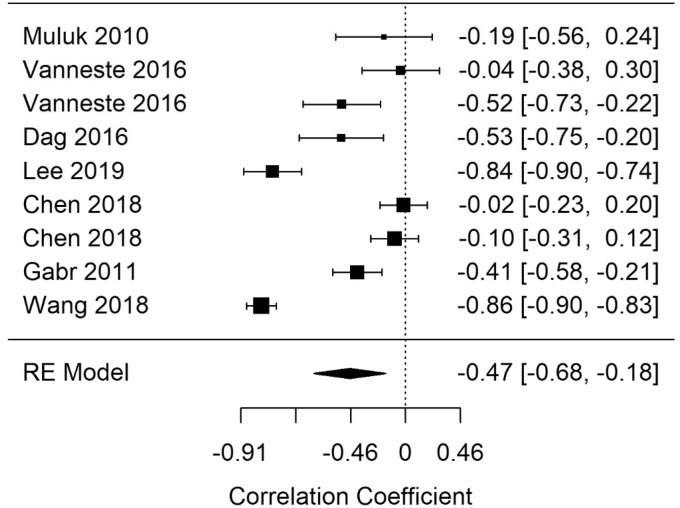
Forest Plot Showing the Correlation Between Tinnitus and Cognitive Screening Measures. Each study is represented by a point estimate bounded by a 95% CI, with the area of each square proportional the study’s weight within the model. Summary effect size is displayed as a polygon at the bottom of the plot, with the width of the polygon representing the 95% CI. RE = Random-effects.

## Supplemental Material

sj-pdf-1-tia-10.1177_2331216520918416 - Supplemental material for Associations Between Subjective Tinnitus and Cognitive Performance: Systematic Review and Meta-Analyses Click here for additional data file. Supplemental material, sj-pdf-1-tia-10.1177_2331216520918416 for Associations Between Subjective Tinnitus and Cognitive Performance: Systematic Review and Meta-Analyses by Nathan A. Clarke, Helen Henshaw, Michael A. Akeroyd, Bethany Adams and Derek J. Hoare in Trends in Hearing

sj-pdf-2-tia-10.1177_2331216520918416 - Supplemental material for Associations Between Subjective Tinnitus and Cognitive Performance: Systematic Review and Meta-Analyses Click here for additional data file. Supplemental material, sj-pdf-2-tia-10.1177_2331216520918416 for Associations Between Subjective Tinnitus and Cognitive Performance: Systematic Review and Meta-Analyses by Nathan A. Clarke, Helen Henshaw, Michael A. Akeroyd, Bethany Adams and Derek J. Hoare in Trends in Hearing

sj-pdf-3-tia-10.1177_2331216520918416 - Supplemental material for Associations Between Subjective Tinnitus and Cognitive Performance: Systematic Review and Meta-Analyses Click here for additional data file. Supplemental material, sj-pdf-3-tia-10.1177_2331216520918416 for Associations Between Subjective Tinnitus and Cognitive Performance: Systematic Review and Meta-Analyses by Nathan A. Clarke, Helen Henshaw, Michael A. Akeroyd, Bethany Adams and Derek J. Hoare in Trends in Hearing

## Appendix: WoE Tool

Evidence is graded according to broad domains of Methodological Quality (WoE A), Methodological Relevance (WoE B), and Topic Relevance (WoE C). Considered domains are given low, medium, or high ratings to provide a final total score of study bias and quality (WoE D).
