# Supplementary material for: Associations Between Subjective Tinnitus and Cognitive Performance: Systematic Review and Meta-Analyses
Source: Trends Hear. 2020 May 21;24:2331216520918416. doi: 10.1177/2331216520918416 (PMC7243410; doi:10.1177/2331216520918416)
Supplement: sj-pdf-1-tia-10.1177_2331216520918416 - Supplemental material for Associations Between Subjective Tinnitus and Cognitive Performance: Systematic Review and Meta-Analyses [file sj-pdf-1-tia-10.1177_2331216520918416.pdf]

Supplemental figures

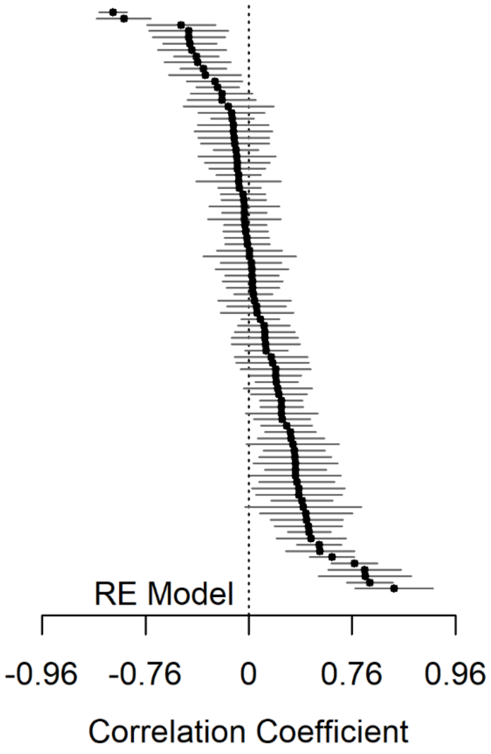

Supplemental figure 1. Caterpillar plot providing overview of effect sizes included in this meta-analysis. Each study included in the meta-analysis is represented by a point estimate, which is bounded by a 95% CI. This plot shows the range of associations across the tinnitus and cognitive performance literature. The plot highlights that large study effect sizes (i.e. correlations > 0.5) should not be viewed as typical. The plot also aims to provide intuitive conceptual value for interested readers who are not familiar with meta-analyses, as they may imagine the initial ‘pile’ of effect sizes being distributed for analysis across the various domains of cognition.
