# Supplementary material for: Associations Between Subjective Tinnitus and Cognitive Performance: Systematic Review and Meta-Analyses
Source: Trends Hear. 2020 May 21;24:2331216520918416. doi: 10.1177/2331216520918416 (PMC7243410; doi:10.1177/2331216520918416)
Supplement: sj-pdf-2-tia-10.1177_2331216520918416 - Supplemental material for Associations Between Subjective Tinnitus and Cognitive Performance: Systematic Review and Meta-Analyses [file sj-pdf-2-tia-10.1177_2331216520918416.pdf]

# Search activity

| | | | | |
|---|---|---|---|---|
| My research question: | Is subjective tinnitus associated with cognitive performance? | | | |
| Places to search for information: | PubMed; B.Ovid MEDLINE; C. Ovid EMBASE; D. PsycINFO; E. ASSIA; F. EBSCO; G. CINAHAL; H. Scopus; I. Web of Science (Science and Social Science Citation Index). | | | |
| | | | | |
| List of sources searched: | Date of search | Search strategy used, including any limits | Total number of results found | Comments |
| PubMed | 14/02/18 | (“tinnitus”[MeSH] OR “tinnitus”[tiab] OR “phantom sound*”[tiab] OR “ringing”[tiab] OR “buzzing”[tiab]) AND (“cognition”[MeSH] OR “cogniti*”[tiab] OR “memory”[tiab] or “attention*”[tiab] OR “executive”[tiab]) | 409 | Limited to human studies |
| PsycINFO (OVID SP) | 14/02/18 | S1: exp TINNITUS/ or phantom sound*.ab. or ringing.ab. or buzzing.ab. or phantom sound*.ti. or ringing.ti. or buzzing.ti.<br><br>S2: exp COGNITION/ or cogniti*.ab. or memory.ab. or attention*.ab. or executive*.ab. or | 336 | |

| | | | | |
|---|---|---|---|---|
| | | cogniti*.ti. or memory.ti. or attention*.ti. or executive*.ti. | | |
| Embase (OVID SP) | 15/02/18 | S1: exp TINNITUS/ or phantom sound*.ab. or ringing.ab. or buzzing.ab. or phantom sound*.ti. or ringing.ti. or buzzing.ti.<br><br>S2: exp COGNITION/ or cogniti*.ab. or memory.ab. or attention*.ab. or executive*.ab. or cogniti*.ti. or memory.ti. or attention*.ti. or executive*.ti. | 2730<br><br>With MedLine excluded: | Embase includes Medline records from 1996 onwards. However if you have already searched PubMed, you may wish to exclude Medline results from your search in Embase<br><br>To limit your search to records unique to Embase add the following limit to your search<br><br>**AND [embase]/lim**<br><br>**This will include records for articles indexed by BOTH Embase and Medline, but exclude articles indexed only for Medline**<br><br>**NOT [medline]/lim**<br><br>**This will exclude ALL Medline records (including articles indexed by BOTH Embase and Medline** |
| Medline 1946-current (OVID SP) | 14/02/18 | S1: exp TINNITUS/ or phantom sound*.ab. or ringing.ab. or buzzing.ab. or phantom sound*.ti. or ringing.ti. or buzzing.ti.<br><br>S2: exp COGNITION/ or cogniti*.ab. or memory.ab. or attention*.ab. or executive*.ab. or | 492 | |

| | | | | |
|---|---|---|---|---|
| | | cogniti*.ti. or memory.ti. or attention*.ti. or executive*.ti. | | |
| ASSIA (via ProQuest) | 14/02/18 | (tinnitus OR "phantom sound" OR ringing OR buzzing) AND (cognition OR cogniti* OR memory OR attention* OR executive*) | 582 | |
| CINAHL plus (via EBSCOhost) | 14/02/18 | ((MH "Tinnitus") OR tinnitus OR ringing OR phantom sound) AND ((MH "Cognition") OR cognition OR cogniti* OR memory OR attention* OR executive*) | 393 | |
| Scopus | 15/02/18 | ( TITLE-ABS-KEY ( tinnitus ) OR TITLE-ABS-KEY ( ringing ) OR TITLE-ABS-KEY ( buzzing ) OR TITLE-ABS-KEY ( "phantom sound" ) AND TITLE-ABS-KEY ( cognition ) OR TITLE-ABS-KEY ( cogniti* ) OR TITLE-ABS-KEY ( memory ) OR TITLE-ABS-KEY ( attention* ) OR TITLE-ABS-KEY ( executive ) OR TITLE-ABS-KEY ( training ) ) AND NOT INDEX ( medline ) | 2261<br><br>974 with not MedLine<br><br>Additional limits: English articles and doctype (article or in-press): 458 | |

| | | | | |
|---|---|---|---|---|
| Web of Science (SSCI) | 1/4/02/18 | (TS=tinnitus OR Ts="ringing" OR Ts="buzzing" OR Ts="phantom sound") AND (TS=cognition OR Ts=cogniti* OR Ts=attention* OR Ts=memory OR Ts=executive*) | 329 | |
