# Supplementary material for: Associations Between Subjective Tinnitus and Cognitive Performance: Systematic Review and Meta-Analyses
Source: Trends Hear. 2020 May 21;24:2331216520918416. doi: 10.1177/2331216520918416 (PMC7243410; doi:10.1177/2331216520918416)
Supplement: sj-pdf-3-tia-10.1177_2331216520918416 - Supplemental material for Associations Between Subjective Tinnitus and Cognitive Performance: Systematic Review and Meta-Analyses [file sj-pdf-3-tia-10.1177_2331216520918416.pdf]

Search activity

My research question:	Is subjective tinnitus associated with cognitive performance?
Places to search for information:	PubMed; B.Ovid MEDLINE; C. Ovid EMBASE; D. PsycINFO; E. ASSIA; F. EBSCO; G. CINAHAL; H. Scopus; I. Web of Science (Science and Social Science Citation Index).

List of sources searched:	Date of search	Search strategy used, including any limits	Total number of results found	Comments
PubMed	25/01/19	(“tinnitus”[MeSH] OR “tinnitus”[tiab] OR “phantom sound*”[tiab] OR “ringing”[tiab] OR “buzzing”[tiab]) AND (“cognition”[MeSH] OR “cogniti*”[tiab] OR “memory”[tiab] or “attention*”[tiab] OR “executive”[tiab])	11	Date limited: 01/02/18<br>Limited to human studies
PsycINFO (OVID SP)	25/01/19	S1: exp TINNITUS/ or phantom sound*.ab. or ringing.ab. or buzzing.ab. or phantom sound*.ti. or ringing.ti. or buzzing.ti.<br><br>S2: exp COGNITION/ or cogniti*.ab. or memory.ab. or attention*.ab. or executive*.ab. or	16	Limit publication year: 2018 to current

		cognitiˆ.ti. or memory.ti. or attention*.ti. or executive*.ti.		
Embase (OVID SP)	25/01/19	S1: exp TINNITUS/ or phantom sound*.ab. or ringing.ab. or buzzing.ab. or phantom sound*.ti. or ringing.ti. or buzzing.ti.<br><br>S2: exp COGNITION/ or cogniti*.ab. or memory.ab. or attention*.ab. or executive*.ab. or cogniti*.ti. or memory.ti. or attention*.ti. or executive*.ti.	55<br><br>2018 to current limit With MedLine excluded:	limit 3 to exclude medline journals<br><br>The above syntax limit applied to the combined search removes the MedLine indexed records that are covered by the PubMed Search
Medline 1946-current (OVID SP)	25/01/19	S1: exp TINNITUS/ or phantom sound*.ab. or ringing.ab. or buzzing.ab. or phantom sound*.ti. or ringing.ti. or buzzing.ti.<br><br>S2: exp COGNITION/ or cogniti*.ab. or memory.ab. or attention*.ab. or executive*.ab. or cogniti*.ti. or memory.ti. or attention*.ti. or executive*.ti.	14	Limit publication year: 2018 to current
ASSIA (via ProQuest)	25/01/19	(tinnitus OR "phantom sound" OR ringing OR buzzing) AND (cognition OR cogniti* OR memory	32	Limit publication year: 02/2018 to current

		OR attention* OR executive*)		
CINAHL plus (via EBSCOhost)	25/01/19	((MH "Tinnitus") OR tinnitus OR ringing OR phantom sound) AND ((MH "Cognition") OR cognition OR cogniti* OR memory OR attention* OR executive*)	56	Limit publication year: 2018 to current
Scopus	25/01/19	(TITLE-ABS-KEY (tinnitus) OR TITLE-ABS-KEY (ringing) OR TITLE-ABS-KEY (buzzing) OR TITLE-ABS-KEY ("phantom sound") AND TITLE-ABS-KEY (cognition) OR TITLE-ABS-KEY (cogniti*) OR TITLE-ABS-KEY (memory) OR TITLE-ABS-KEY (attention*) OR TITLE-ABS-KEY (executive) OR TITLE-ABS-KEY (training)) AND NOT INDEX (medline)	108 with not MedLine<br><br>Additional limits: doctype (article or in-press): 79	Limit publication year: 02/2018 to current
Web of Science (SSCI)	25/01/19	(TS=tinnitus OR Ts="ringing" OR Ts="buzzing" OR Ts="phantom sound") AND (TS=cognition OR Ts=cogniti* OR Ts=attention* OR Ts=memory OR Ts=executive*)	53	Limit publication year: 2018 to current
